# Portable and Reusable Optofluidics-Based Biosensing Platform for Ultrasensitive Detection of Sulfadimidine in Dairy Products

**DOI:** 10.3390/s150408302

**Published:** 2015-04-09

**Authors:** Xiu-Juan Hao, Xiao-Hong Zhou, Yan Zhang, Feng Long, Lei Song, Han-Chang Shi

**Affiliations:** 1 State Key Joint Laboratory of ESPC, School of Environment, Tsinghua University, Beijing 100084, China; E-Mails: hxq_66@126.com (X.-J.H.); hanchang@mail.tsinghua.edu.cn (H.-C.S.); 2 Civil Engineering Institute, Inner Mongolia University of Technology, Hohhot 010051, China; E-Mail: nngsong@126.com (L.S.); 3 Hebei Institute of Food Quality Supervision Inspection and Research, Shijiazhuang 050091, China; E-Mail: snowwinglv@126.com; 4 School of Environment and Natural Resources, Renmin University of China, Beijing 100872, China; E-Mail: longf04@mails.tsinghua.edu.cn

**Keywords:** optofluidics-based biosensing platform, optic fiber, sulfadimidine, dairy products

## Abstract

Sulfadimidine (SM_2_) is a highly toxic and ubiquitous pollutant which requires rapid, sensitive and portable detection method for environmental and food monitoring. Herein, the use for the detection of SM_2_ of a portable optofluidics-based biosensing platform, which was used for the accurate detection of bisphenol A, atrazine and melamine, is reported for the first time. The proposed compact biosensing system combines the advantages of an evanescent wave immunosensor and microfluidic technology. Through the indirect competitive immunoassay, the detection limit of the proposed optofluidics-based biosensing platform for SM_2_ reaches 0.05 μg·L^−1^ at the concentration of Cy5.5-labeled antibody of 0.1 μg·mL^−1^. Linearity is obtained over a dynamic range from 0.17 μg·L^−1^ to 10.73 μg·L^−1^. The surface of the fiber probe can be regenerated more than 300 times by means of 0.5% sodium dodecyl sulfate solution (pH = 1.9) washes without losing sensitivity. This method, featuring high sensitivity, portability and acceptable reproducibility shows potential in the detection of SM_2_ in real milk and other dairy products.

## 1. Introduction

Sulfadimidine (SM_2_) is an antibiotic which is widely used in human and veterinary medicine for effective treatment and prevention of diseases, or as growth promoter of farm animals, e.g., cows [1]. Its extensive use and high rate of pharmaceutical consumption can lead to the appearance of residues in water via the effluents and products of animal origin. It causes serious side effects such as hypersensitive allergic reactions, drug-resistance problems in human and, even carcinogenic effects [2,3,4,5]. For protection of human health, strict maximum residue limits for the total sulfonamide residue in foods of 100 μg·L^−1^ have been set by European regulatory authority and Chinese government [6,7,8]. Quantifying SM_2_ residue levels in the food samples (e.g., milk and other dairy products) is therefore of paramount importance.

Many analytical methods based on different devices, such as high performance liquid chromatography (HPLC) [9,10], liquid chromatography–mass spectrometry (LC–MS) [11,12,13,14,15], ELISA [16,17], have been reported for SM_2_ monitoring. The abovementioned methods are both sensitive and selective for quantitative analysis of SM_2_, but require sophisticated instrumentation, complex extraction steps and time-consuming operation procedures. Therefore, developing a detection method for SM_2_, which is not only sensitive and selective, but also simple, portable and cost-effective in its operation, is required in food and environmental monitoring.

Immunosensor detection is a powerful method widely used in the detection of small molecule pollutants at trace concentrations [18,19]. Combining this technique with evanescence wave technology enables the implementation of sensitive, selective, portable and cost-effective immunoassays [20,21]. Moreover, the evanescent wave immunosensor is regarded as one of the most important optofluidic technologies [22,23,24]. Herein we report an ultrasensitive and reusable optofluidics-based biosensing platform based on an evanescent wave immunosensor and microfluidic technology. This is the first application of these combined techniques to the analysis of trace amounts of SM_2_ and their analysis in milk and other dairy products. The evanescence wave immunosensor system together with the microfluidic technology can meet the requirements of *in-situ* screening. Compared with our previous system (using a fiber probe of 85 mm in length), the new dimensions were decreased to be 40 mm in length, which was favorable due to the advantages of a microfluidic system, e.g., modest cost, and the dimension change was proved to have no effect on the assay results. This system adopts a single-multi mode fiber optic coupler to achieve transmission of excitation light, and collection and transmission of the generated fluorescence. A multi-mode fiber probe is made sensitive to the target by the covalent immobilization of the hapten conjugate of SM_2_-bovine serum albumin (SM_2_-BSA). Excitation light is propagated in the probe via the total internal reflection mode to form an evanescent field on the fiber surface, which can excite fluorescent molecules attached on the surface. Through an indirect competitive immunoassay, the fluorescence intensity is inversely related with SM_2_ concentrations in the test samples. The proposed straightforward method can fully facilitate the measurement of SM_2_ and meet the stringent demands of applications in food and environmental monitoring.

## 2. Experimental Section

### 2.1. Apparatus

**Figure 1:**
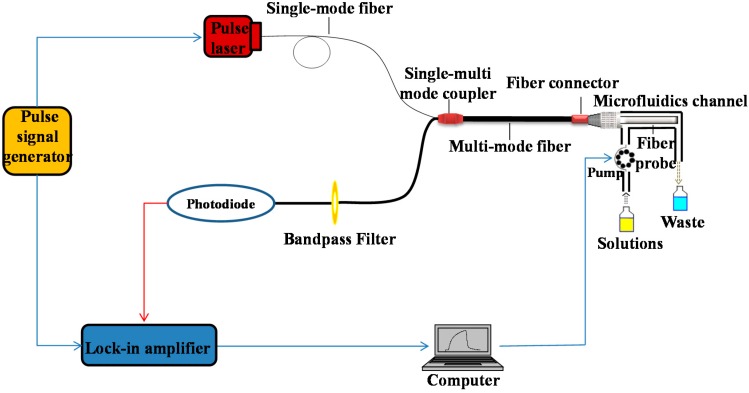
Schematic set-up of our portable and reusable optofluidics-based biosensing platform.

The optofluidics-based biosensing platform applied in this study has been described in detail in the [22] and is presented in Figure 1. Briefly, the platform comprises three parts: a microfluidics system, a fiber-optic biosensor system and a built-in computer. The control of the fluid delivery system, data acquisition and processing were automatically performed by the built-in computer. All reagents were delivered to a microfluidics channel made of poly(methyl methacrylate) (PMMA) by a flow delivery system operated by a peristaltic pump. The plastic-clad step-index silica optical fiber (core diameter of 600 μm and NA of 0.22) was embedded into the microfluidics channel with effective dimensions of 40 mm in length and 600 µm in the surrounding thickness of the fiber. The pulse laser beam from a 635-nm pulse diode laser was directly launched with a pigtail into a multi-mode fiber probe through the single-multi mode fiber coupler. The incident light propagates along the length of the probe via total internal reflection. The evanescent wave generated at the surface of probe then interacted with the surface-bound fluorescently-labeled target conjugate, and causes excitation of the fluorophores. The collected fluorescence was transmitted back through the fiber probe, and subsequently filtered by a band pass filter and detected by a lock-in amplifier. Details of the fabrication and preparation of the combination tapered fiber probe can be found in [25,26].

### 2.2. Chemicals and Reagents

(3-Aminopropyl) triethoxysilane (APTES), glutaraldehyde (GA), bovine serum albumin (BSA), sodium dodecyl sulfate (SDS) and sulfadimidine were purchased from Sigma-Aldrich (Shanghai, China). SM_2_ stock solution (1 mg·mL^−1^) was purchased from Putian Tongchuang Biotechnology Co., Ltd., (Beijing, China). Other reagents, if not specified, were supplied by Beijing Chemical Agents (Beijing, China). All reagents were of analytical grade and used without further purification. Deionized water was used throughout the experiments. Standard concentrations of the target were prepared from the stock solution by serial dilutions in 0.01 M phosphate buffer solutions (PBS, pH = 7.4, 137 mM NaCl + 2.7 mM KCl + 4.3 mM Na_2_HPO_4_ + 1.4 mM KH_2_PO_4_). The SM_2_ monoclonal antibody and hapten conjugate of SM_2_ and carrier protein was purchased from Shijiazhuang Solarpex Biotechnology Co., Ltd. (Shijiazhuang, China) and labeled with Cy 5.5 (GE Healthcare Life Sciences, Shanghai, China) according to the procedure proposed by Mujumdar *et al.* [27].

### 2.3. Surface Chemical Modification of Optic Fiber Probe

In order to specifically combine the probe with the Cy5.5-labeled antibody, the hapten conjugate of SM_2_ and carrier protein were immobilized covalently on the unclad region to form a biosensitive probe [28,29,30] as follows: prior to surface modification, the probe was soaked in a 3:7 (v/v) mixture of 30% H_2_O_2_/98% H_2_SO_4_ an hour for hydroxylation, followed by thorough rinsing with ultrapure water and drying in an oven at 120 °C. Then, the fiber probe was immersed in 2% (v/v) APTES toluene solution for 1 h at room temperature in an attempt to form amino groups on the fiber probe surface. Subsequently, the fiber probe was washed with dry toluene to remove excess APTES on the surface and dried in an oven for 30 min at 100 °C. To immobilize SM_2_-BSA onto the surface of the amino-silanized probe, the fiber modified with amino groups was allowed to react with a heterobifunctional crosslinker, 2.5% GA solution, for 1 h at 37 °C, and thoroughly washed with PBS buffer. After that, the fiber probe was immersed overnight in 0.02 μg·mL^−1^ SM_2_-BSA solution in PBS buffer at 4 °C. Ultimately, the fiber probe was soaked in 2 mg·mL^−1^ BSA for 1 h at room temperature to block any remaining non-specific binding sites. The modified fiber probe was stored at 4 °C before use.

### 2.4. Immunoassay Procedures and Regeneration

**Figure 2:**
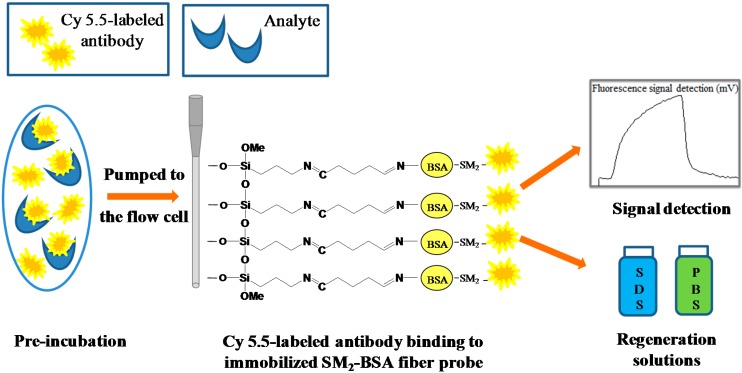
Schematic of sensing mechanism for SM_2_ detection based on evanescent wave optofluidics-based biosensing platform.

Different concentrations of SM_2_ standard solutions ranging from 0.01 to 1000 μg·L^−1^ were prepared in 0.01 M PBS. The Cy5.5-labeled antibody at concentrations 0.1 μg·mL^−1^ and 0.4 μg·mL^−1^ were chosen for SM_2_ measurement experiments. For the indirect competitive assay as shown in Figure 2, standard solutions containing various concentrations of SM_2_ were mixed with a fixed amount of Cy5.5-labeled antibody for 5 min (pre-incubation) at 37 °C, then the mixture was pumped through the microfluidic channel for 1 min at a rate of 400 μL·min^−1^ for 5 min, and the fluorescence signal was recorded in real-time. To regenerate the probe surface, the probe surface was alternately washed for 1 min by using regeneration solutions 0.5% SDS buffer (pH = 1.9) and 0.01 M PBS buffer for two cycles. All experiments were performed at room temperature if not specified otherwise.

### 2.5. Recovery Experiments

Dairy product samples of liquid milk, yoghourt and baby formula milk, were bought from a local supermarket. For solid baby formula milk, 4 g of sample was dissolved in 20 mL 0.01 M PBS buffer at 80 °C for 5 min. Then, 2 mL dissolved formula milk (or raw liquid milk/yoghourt), 3 mL acetonitrile and 15 mL 10% trichloroacetic acid were added into a centrifuge tube and then spiked with SM_2_ standard solutions to final concentrations at both levels of 0.5 μg·L^−1^ and 1 μg·L^−1^. The mixture was centrifuged at 12,000 rpm for 5 min to precipitate protein and dissolve organic substances. Subsequently, 200 μL of the supernatant sample was diluted with 0.01 M PBS to 10 mL for optofluidics-based biosensing platform detection. 

### 2.6. Data Analysis

The standard curves were plotted against the logarithm of concentration of SM_2_ through a five-parameter logistic model as follows [31]:

$$\text{SS}=\;\frac{A_{1}-A_{2}}{1+{\left([\text{Ag}]/[{\text{Ag}}_{0}]\right)}^{p}}+A_{2}$$

Herein [Ag] is the SM_2_ concentration; SS is the signal strength of optofluidics-based biosensing platform; A_1_ and A_2_ are the maximum (blank signal, *x*→0) and minimum signal (background signal, *x*→∞) to the titration curve; [Ag_0_] is the target concentration at the midpoint or inflection point (IC_50_); and *p* is the slope of the tangent at the inflection point. The 50% inhibition values of the cross-reactivity (CR) were used to judge the selectivity of the sensing system via the following formula [32]:

$$\text{CR}\left(\%\right)=[{\text{IC}}_{50}({\text{SM}}_{2})/{\text{IC}}_{50}(\text{structural}\;\text{analogue})]\times 100\text{\%}$$

## 3. Results and Discussion

### 3.1. Analytical Performance

**Figure 3:**
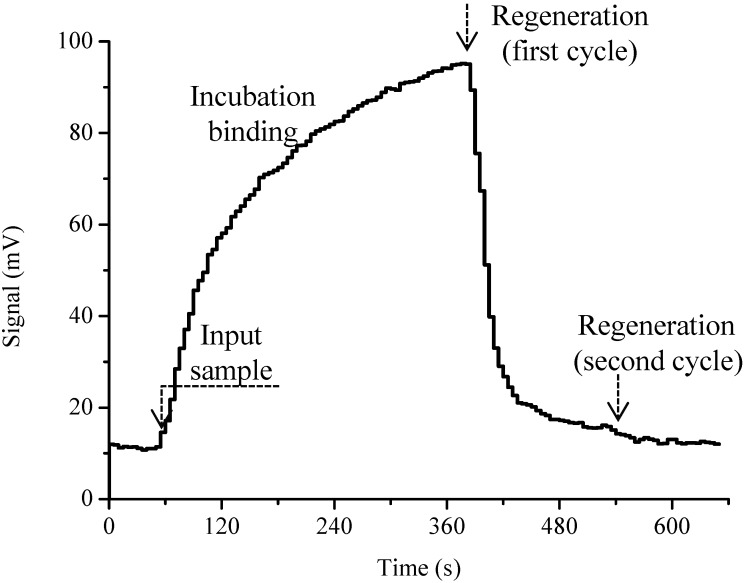
Typical signal trace for SM_2_ determination on the optofluidics-based biosensing platform during a whole test cycle.

In the proposed optofluidics-based biosensing platform, an indirect competitive immunoassay was adopted. SM_2_–BSA was immobilized on the fiber probe surface to compete with SM_2_ target in the test samples to bind the Cy5.5-labeled SM_2_ antibody. The fluorescent signals caused by the binding of Cy5.5-labeled antibody would be reversely related with the analyte concentration in the test sample. The signal trace for a whole test cycle for SM_2_ determination is shown in Figure 3. On the platform of proposed optofluidics-based biosensing system, no more than 15 min were needed for one assay cycle including 5 min pre-incubation.

**Figure 4:**
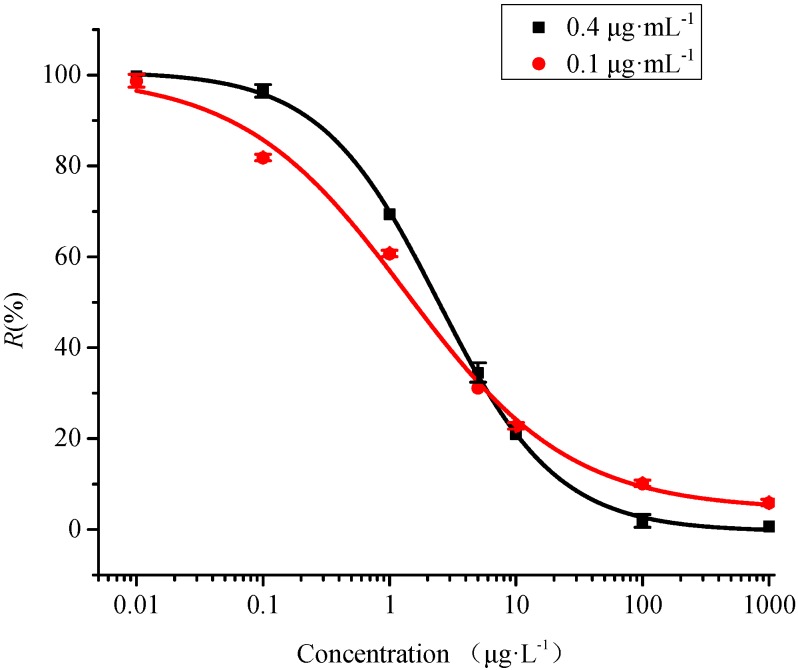
Standard curves of SM_2_ determination by optofluidics-based biosensing platform, respectively, at two different Cy5.5-labeled antibody concentrations of 0.1 μg·mL^−1^ and 0.4 μg·mL^−1^.

Figure 4 shows the representative competitive inhibition curves for SM_2_ at the Cy5.5-labeled antibody concentrations of 0.1 μg·mL^−1^ and 0.4 μg·mL^−1^. For SM_2_ detection, all the signals were presented by average value and standard deviation (S.D.) of three times of measurements as follows:

$$R(\%)=\frac{S}{S_{0}}$$

where R is the normalized ratio, S is the fluorescence signal (when the SM_2_ concentration is given), S_0_ is the maximum fluorescence signal (containing zero SM_2_ concentration). The fluorescence signals were recorded for SM_2_ concentrations between 0.01 μg·L^−1^ and 1000 μg·L^−1^ in PBS buffer (pH 7.4). The amount of Cy5.5-labeled antibodies binding to the fiber probe decreased with increasing SM_2_ concentration in the mixture, causing the fluorescence to gradually decrease, which was in complete agreement with the decrease in the free Cy5.5-labeled antibody left. When the target concentrations were higher than 1000 μg·L^−1^, no fluorescence signal could be observed. This confirmed without doubt that the fluorescence signal obtained was not due to non-specific adsorption of Cy5.5-labeled antibodies in the solution.

As shown in Figure 4, the linear quantitative SM_2_ detection range of the optofluidics-based biosensing platform ranged from 0.17 μg·L^−1^–10.73 μg·L^−1^ (0.1μg·mL^−1^ Cy5.5-labeled antibody) and 0.54 μg·L^−1^–10.85 μg·L^−1^ (0.4 μg·mL^−1^ Cy5.5-labeled antibody) described by 20%–80% inhibitory concentrations. The limit of detection (LOD) defined as the target concentration providing a 10% decrease of the A_1_ was also determined to be 0.05 μg·L^−1^ at 0.1 μg·mL^−1^ Cy5.5-labeled antibody and 0.23 μg·L^−1^ at 0.4 μg·mL^−1^ Cy5.5-labeled antibody, respectively. For comparison, the LOD calculated by definition as the concentration required to give a signal equal to the blank signal plus three times the standard deviations of the blank, were 0.07 μg·L^−1^ at 0.1 μg·mL^−1^ Cy5.5-labeled antibody and 0.31 μg·L^−1^ at 0.4 μg·mL^−1^ Cy5.5-labeled antibody, which were close to the abovementioned LOD defined as the concentration where the normalized signal value was lower by 10%. In order to match the normalized signal values in Figure 4, the first mentioned LOD value was adopted in this study. The LOD values of the sensor at two different antibody concentrations were more sensitive compared with the maximum detection limit of SM_2_ in food imposed by the European regulatory authority and Chinese government [6,7,8]. Our results also demonstrated that the concentration of Cy5.5-labeled antibody did affect the accuracy and linear range of the proposed biosensing platform as in previous studies [22]. The Cy5.5-labeled antibody concentration of 0.1 μg·mL^−1^ featured both higher detection sensitivity and reduced reagent costs. Therefore, 0.1 μg·mL^−1^ Cy5.5-labeled antibody concentration was adopted in the experiment. Compared with other methods reported previously, such as HPLC (5–6.4 μg·L^−1^) [9,10], LC-MS (3 μg·L^−1^) [11] and ELISA (0.15 μg·L^−1^) [17], our results provided a higher sensitivity for SM_2_ detection with the lowest LOD.

### 3.2. Selectivity

Cross-reactivity is an important analytical parameter regarding the specificity and reliability of immunoassays [33]. Selectivity of the proposed biosensing platform was investigated by assessing the cross-reactivity of this method by measuring the inhibition curves of three structural analogues, e.g., sulfamerazine (SMR), sulfamethoxazole (SMX), and sulfadiazine (SDZ) as competitors. Table 1 shows that SMR, SMX, SDZ exhibited low cross-reactivity (CR < 0.08%) toward the antibody, although very similar pollutant molecules were applied. We attribute the high selectivity to the specificity of Cy5.5-labeled SM_2_ antibody, which is an important factor that affects the rapid assessment of pollutant molecules without separation in food monitoring. The results confirmed that the proposed biosensor system was barely affected by other structural analogues and indicated that the proposed optofluidics-based biosensing platform appears less susceptible to interference from other closely resembling food and environmental pollutant molecules during SM_2_ determination.

**Table 1:** Cross-reactivities of anti-sulfadimidine (SM_2_) antibody based on optofluidics-based biosensing platform towards sulfamerazine (SMR), sulfamethoxazole (SMX) and sulfadiazine (SDZ) in buffer solutions.

Structurally Analogues	IC_50_ μg·L^−1^	LOD μg·L^−1^	CR %
SM_2_	0.05	0.05	100
SMR	60.08	14.41	<0.08
SMX	>10000	-	<10^−5^
SDZ	>10000	-	<10^−5^

### 3.3. Reproducibility and Stability

**Figure 5:**
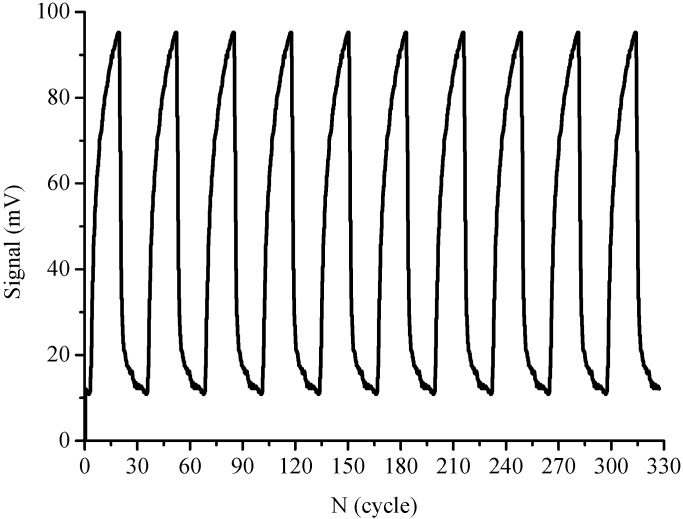
Regeneration of immunoassay signals by means of optofluidics-based biosensing platform with influent of 0.1 μg·mL^−1^ Cy5.5-labeled antibody.

In the assays described in this work regeneration and the sensing surface binding properties of the sensor were also the major concerns in evaluating the immunosensor; these completely depend on the regeneration capabilities and accuracy of detection results [34]. In this study; 0.5% SDS solution (pH 1.9) was chosen as regeneration solution for regeneration of the sensor surface without damage to its physical-chemical properties; which is essential for repeated use of the sensor probe. After each SM_2_ determination cycle; there was no obvious change in the baseline value and surface binding capability after regeneration, implying that no apparent chemical damages occurred in the hapten-protein conjugate layer. The complete removal of non-covalently bound Cy5.5-labeled antibody for SM_2_-BSA conjugates was confirmed. Since the signal returned to the baseline after regeneration, further injection of the same concentration of Cy5.5-labeled antibody gave an equivalent augmented signal (Figure 5). 

After 300 regenerated assays conducted over three months, the fiber probe could still be used for detection of SM_2_ without much loss of sensitivity (RSD = 1.20%). During the three months, the fiber probe was kept at 4 °C after a whole day of experiments until the next measurement. Therefore, it can be deduced that the fiber probe coated with SM_2_-BSA conjugates can be stored more than 3 months at 4 °C. The robustness and stability of the hapten conjugates immobilized on the probe surface is one of the distinct advantages of the proposed biosensing platform over other detection methods (e.g., ELISA formats) [35,36], also indicating that the proposed method is cost-effective and reliable for SM_2_ measurement.

### 3.4. Detection of SM_2_ in Real Milk and Other Dairy Samples

To evaluate the accuracy and potential applications of the proposed biosensing platform for detection of SM_2_ residues in food, three spiked samples of milk and other dairy products including liquid milk, yoghurt, baby formula, were assayed. The fluorescence signals were almost the same as the response to the buffer solution before the real samples were spiked with SM_2_ for milk and other dairy products, indicating the initial concentrations of SM_2_ in test samples were negligible. The recovery ratios (Table 2) measured by the proposed biosensing platform were calculated in the range from 97% to 116%. Satisfactory variations were also demonstrated as the RSD values were no more than 0.71%. These results indicate that the proposed biosensing platform is suitable for the application to SM_2_ detection in real milk and other dairy samples.

**Table 2:** Recovery of SM_2_ by means of optofluidics-based biosensing platform in milk and other dairy products (*n* = 3).

Sample	Spiked μg·L^−^^1^	Measured μg·L^−^^1^	RSD (%)	Recovery (%)
Milk	0.50	0.49	0.35	97.82
	1.00	1.00	0.71	99.78
Yoghurt	0.50	0.53	0.35	105.44
	1.00	1.16	0.71	115.94
Baby formula	0.50	0.55	0.71	109.47
	1.00	1.08	0.35	107.56

## 4. Conclusions

In this study, we have introduced a simple and rapid optofluidics-based biosensing platform for SM_2_ determination. The optofluidics-based biosensing platform showed superior selectivity and regeneration capability toward SM_2_. This is due to the surface chemical modification of SM_2_-BSA conjugates on the probe surface, which not only maintained the activities of the immobilized SM_2_ towards antibodies, but also cut down the non-specific adsorption by immersing the fiber surface in the BSA. The whole test cycle including the regeneration process needs less than 15 min. A wide linear dynamic detection range from 0.17 μg·L^−1^ to 10.73 μg·L^−1^ with a detection limit of 0.05 μg·L^−1^ is obtained, which satisfies the needs for rapid monitoring of trace SM_2_ levels in milk and other dairy products. Furthermore, the acceptable cross-reactivity towards structural analogues of SM_2_, e.g., SMR, SMX, and SDZ was observed. SM_2_ recoveries in the range from 97% to 116% were achieved in spiked milk and other dairy samples. Overall, the optofluidics-based biosensing platform is a promising tool for rapid, ultrasensitive, selective, portable and cost-effective detection of SM_2_ in food and environmental monitoring. With all the abovementioned advantages, the proposed optofluidics-based biosensing platform is expected to be practically applied in many fields where detection of SM_2_ is of prime concern.
